# Allometric relationships for eight species of 4–5 year old nitrogen-fixing and non-fixing trees

**DOI:** 10.1371/journal.pone.0289679

**Published:** 2023-08-21

**Authors:** K. A. Carreras Pereira, Amelia A. Wolf, Sian Kou-Giesbrecht, Palani R. Akana, Jennifer L. Funk, Duncan N. L. Menge

**Affiliations:** 1 Department of Ecology, Evolution and Environmental Biology, Columbia University, New York, New York, United States of America; 2 Department of Integrative Biology, University of Texas Austin, Austin, Texas, United States of America; 3 Canadian Centre for Climate Modelling and Analysis, Victoria, British Columbia, Canada; 4 Department of Earth and Environmental Sciences, Dalhousie University, Halifax, Nova Scotia, Canada; 5 Department of Plant Sciences, University of California, Davis, Davis, California, United States of America; ICAR Research Complex for Eastern Region, INDIA

## Abstract

Allometric equations are often used to estimate plant biomass allocation to different tissue types from easier-to-measure quantities. Biomass allocation, and thus allometric equations, often differs by species and sometimes varies with nutrient availability. We measured biomass components for five nitrogen-fixing tree species (*Robinia pseudoacacia*, *Gliricidia sepium*, *Casuarina equisetifolia*, *Acacia koa*, *Morella faya*) and three non-fixing tree species (*Betula nigra*, *Psidium cattleianum*, *Dodonaea viscosa*) grown in field sites in New York and Hawaii for 4–5 years and subjected to four fertilization treatments. We measured total aboveground, foliar, main stem, secondary stem, and twig biomass in all species, and belowground biomass in *Robinia pseudoacacia* and *Betula nigra*, along with basal diameter, height, and canopy dimensions. The individuals spanned a wide size range (<1–16 cm basal diameter; 0.24–8.8 m height). For each biomass component, aboveground biomass, belowground biomass, and total biomass, we determined the following four allometric equations: the most parsimonious (lowest AIC) overall, the most parsimonious without a fertilization effect, the most parsimonious without canopy dimensions, and an equation with basal diameter only. For some species, the most parsimonious overall equation included fertilization effects, but fertilization effects were inconsistent across fertilization treatments. We therefore concluded that fertilization does not clearly affect allometric relationships in these species, size classes, and growth conditions. Our best-fit allometric equations without fertilization effects had the following R^2^ values: 0.91–0.99 for aboveground biomass (the range is across species), 0.95 for belowground biomass, 0.80–0.96 for foliar biomass, 0.94–0.99 for main stem biomass, 0.77–0.98 for secondary stem biomass, and 0.88–0.99 for twig biomass. Our equations can be used to estimate overall biomass and biomass of tissue components for these size classes in these species, and our results indicate that soil fertility does not need to be considered when using allometric relationships for these size classes in these species.

## 1. Introduction

Allometric equations facilitate the estimation of important but labor-intensive tree properties (e.g., total biomass and its components) from easily measured tree properties (e.g., diameter), and therefore are a key tool for ecosystem ecology, forest ecology, forestry, and other fields [1, 2]. Well-calibrated allometric equations are available for numerous species [e.g., 3] but not for many others. They are much more common for aboveground tissues than for belowground tissues and, within aboveground tissues, for total or woody tissues than for foliage or different size classes of woody tissues [4, 5] (but see [6–8]). The relative paucity of biomass data for belowground tissues, foliage, and different size classes of woody tissues likely stems from logistical challenges, but these data are important. For instance, understanding the contribution of roots, which account for a significant fraction of total tree biomass (an average of 20% globally [9]), is critical for quantifying soil carbon stocks [10, 11]. Foliage, twigs, small branches, and main stems have very different nutrient contents [12], so accurately modeling nutrient budgets depends on an ability to estimate them separately.

Theory [13–17] suggests that trees allocate biomass differently as nutrient availability declines. Some studies show that plants allocate more to roots in infertile conditions [8, 16, 17], though the details vary. Allometric relationships within aboveground tissues can also change across nutrient conditions. For example, adaptive dynamics theory predicts more allocation to wood as opposed to foliage as fertility increases [17]. Empirically, allometric relationships vary with nutrient availability for some species, but not all. For example, Urban *et al*. [18] found that Norway spruce trees were shorter for a given diameter in a nutrient-poor site than in a nutrient-rich site, whereas Douglas fir trees had similar height-diameter relationships in both sites.

Trees that form symbioses with nitrogen (N)-fixing bacteria (hereafter, “N-fixing trees”) occupy an interesting role in this discussion. Symbiotic N-fixing trees are commonly planted during reforestation efforts, particularly on marginal soils [19, 20], due to their ability to bring newly fixed N into ecosystems. Their rapid growth on marginal sites provides carbon sequestration [21] and soil regeneration [22–25]. Aside from restoration efforts, N-fixing trees have a history of use in forest plantations and agroforestry to provide fast-growth timber (e.g., *Casuarina*, *Alnus)* [26], and to relieve N limitation by intercropping [27] or in mixed-species tree plantations [28]. Given that they have access to an unlimited N pool (atmospheric N_2_), they are less likely to be limited by N, and thus might be less likely to alter allocation patterns in response to soil N availability. For example, Markham & Zekveld [29] found that increasing soil N availability did not affect root biomass allocation in seedlings of the N-fixing *Alnus viridis*, but that uninoculated (and thus non-fixing) seedlings of the species increased root allocation and had 25% lower total biomass in low N soils. However, not all N-fixing species act similarly: Taylor & Menge [30] found that N fertilization led to lower root allocation for inoculated as well as uninoculated seedlings of *Pentaclethra macroloba*.

Our primary study objective was to establish allometric equations for common N-fixing and non-fixing tree species in the coterminous USA and Hawaii. Given the possible effects of nutrients or functional type (N-fixer vs. non-fixer) on these allometric relationships, we also asked two secondary questions. To what degree does fertilization affect the allometric relationships of 1a) the tree species and 1b) of the functional types (N-fixers and non-fixers)? 2) How do allometric relationships differ between N-fixing and non-fixing trees? For the N-fixing species, we studied *Robinia pseudoacacia*, which is the most abundant N-fixing tree in the coterminous USA [31], and four tropical N-fixing trees that are regionally or locally common: *Gliricidia sepium*, *Acacia koa*, *Casuarina equisetifolia*, and *Morella faya*. For the non-fixing comparison, we chose *Betula nigra*, *Psidium cattleianum*, and *Dodonaea viscosa*, which are common species that co-occur with the N-fixers we studied.

## 2. Methods

### 2.1 Study sites and species

We studied eight fast-growing tree species in one temperate and two tropical sites. At each site, we studied at least one N-fixing and one non-fixing species. Each species was chosen due to widespread regional and/or local abundance. Many of the chosen species also have a long history of use in agroforestry. In Black Rock Forest (41.42° N, 74.02° W, 195 m elevation), New York state, USA, we studied one N-fixing species, *Robinia pseudoacacia* L., and one non-fixing species, *Betula nigra*. Black Rock Forest is an oak-dominated 1550-ha forest preserve [32] with a long history of forest research [33, 34]. It has a temperate climate: mean annual temperature (MAT) and precipitation (MAP) are 9.5°C and 1248 mm y^−1^ [35], respectively, with monthly average temperatures ranging from −2.7°C in January to 23.4°C in July [36, 37]. The soils are medium-textured loams, though glacial till is apparent typically at 0.25 to 1 m [38]. *Robinia* (hereafter, we refer to each species by its genus name alone) is native to eastern North America and is the most abundant N-fixing tree in the United States, accounting for 64% of all tree-based N fixation in the coterminous USA [31]. It is also common throughout Eurasia [39, 40], and is commonly used in plantations [41]. *Betula*, also native to eastern North America, is found growing mainly in wet soil conditions ranging from east Texas to New England [42].

We studied four N-fixing tree species and two non-fixing tree species in two sites on the island of Hawaii. At the University of Hawaii Waiakea Research Station, Hawaii, USA (“Waiakea;” 19.64° N, 155.08° W, 196 m elevation), we studied N-fixing *Gliricidia sepium*, N-fixing *Casuarina equisetifolia*, and non-fixing *Psidium cattleianum*. Waiakea has a tropical climate, with MAT of 23.3°C and MAP of 4,318 mm y^−1^. The soils at Waiakea are classified as Keaukaha extremely rocky muck, a sapric histosol with numerous cobble- and gravel-sized clasts underlain by pahoehoe lava [43]. *Gliricidia* is hypothesized to be native to Pacific/western coasts of Mexico and Central America, where it is commonly used in agroforestry as a live fence and fodder, among other uses [44]. It has been naturalized in many subtropical and tropical environments throughout Asia and Australia as well as Hawaii [45]. *Casuarina*, or the Australian pine, is an invasive N-fixing evergreen angiosperm that grows along coastal dunes, where it is often planted for its ability to grow in sandy, salty soils and its wind-resistant qualities. In agroforestry, *Casuarina* is planted for erosion control and is used in plantations for paper production [46, 47]. *Psidium* is an evergreen tree or shrub native to Brazil that produces a fruit known as the strawberry guava (araçá). *Psidium* is well-adapted to varying climates and grows in dense thickets [48, 49], spreading as a noxious weed throughout islands in the South Pacific and Indian Oceans [50].

At the University of Hawaii Volcano Research Station, Hawaii, USA (hereafter referred to as “Volcano;” 19.47° N, 155.26°W, 1249 m elevation), we studied N-fixing *Acacia koa*, N-fixing *Morella faya*, and non-fixing *Dodonaea viscosa*. Volcano has a montane tropical climate with a MAT and MAP of 16.3°C and 3,048 mm y^−1^ respectively. The soils at Volcano are typic hydrandepts of a puaulu series [51]. *Acacia* is one of the largest and most common forest trees found across the Hawaiian archipelago, used in forest restoration due to its nature as an endemic, rapidly-growing legume [52]. It is also economically valuable as timber [53] and culturally significant for its use in canoe-building [54]. *Morella* is an invasive species first introduced to Hawaii in the 1800s from islands in the Atlantic (Canary, Madeira, and Azores). It is widely distributed, extending south to Australia and New Zealand [55]. Its N-fixing capabilities and dense canopy structure pose a threat to the endemic ecological landscape, as it supplants native shade-intolerant species [56–59]. *Dodonaea* is a tropical and subtropical shrub or small tree ranging from 2-6m in height [60] considered to be native to Hawaii and hypothesized to originate from Australia [60, 61]. Although both *Psidium* and *Dodonaea* can grow as shrubs as well as trees, their growth rate, abundance, and other characteristics made them the best matches for our N-fixing trees among the available options near Waiakea and Volcano, respectively. All three research stations (Black Rock Forest, the University of Hawaii Waiakea Research Station, and the University of Hawaii Volcano Research Station) granted us permission to use the sites. No formal permits were required to conduct our research since all research stations operate the land.

### 2.2 Study design

We planted bare-root seedlings in May 2015 (Black Rock) and May 2016 (Waiakea and Volcano) in open fields. We replaced trees that died within the first year of the experiment but did not replace trees that died in subsequent years. The mean initial basal diameter and height after planting were 0.49 cm and 0.36 m (*Robinia*), 0.52 cm and 0.52 m (*Betula*), 0.67 cm and 0.18 m (*Gliricidia*), 0.30 cm and 0.52 m (*Casuarina*), 0.24 cm and 0.20 m (*Psidium*), 0.35 cm and 0.32 m (*Acacia*), 0.18 cm and 0.095 m (*Morella*), and 0.12 cm and 0.047 m (*Dodonaea*). Wire cages were installed around all trees to minimize damage from large mammals (deer in New York, pigs in Hawaii), and the cages were removed before they would start to affect growth. In New York, we applied glyphosate in the first four years of the experiment to inhibit competition from ground-layer plants. In Hawaii, we applied glyphosate in the first year, but in the following years we mowed and weeded (within 0.5 m of each plant) since the glyphosate contributed to the death of a number of trees.

Each N-fixing tree was matched with a non-fixing tree, either as pairs (one N-fixer, one non-fixer) in New York or as linear triads (two N-fixers, one non-fixer) in Hawaii. Trees in each pair or triad were placed 5 m apart from each other, and each tree was at least 12 m from all trees in other pairs or triads. Each pair or triad received the same fertilization treatment. See [62] for a graphical depiction of the plot layout and further details. We planted 8 (in Black Rock and Volcano) or 9 (in Waiakea) replicate pairs or triads of each of four fertilization treatments: control (hereafter denoted as “C”), +10 g N m^−2^ y^−1^ (hereafter denoted as “+10”), +15 g N m^−2^ y^−1^ (hereafter denoted as “+15”), and +15 g N m^−2^ y^−1^ +15 g P m^−2^ y^−1^ (hereafter denoted as “+15+P”). The control treatment received 0.1 g N m^−2^ y^−1^ as ammonium nitrate in years 2 and 3 and none in all other years. This small addition of ammonium nitrate was isotopically labeled in order to facilitate the measurement of symbiotic N fixation, which was a major goal of the overall experiment [63], though not our focus here. As is standard in the enriched isotopic pool dilution method of measuring symbiotic N fixation [64], we needed to add a small amount (0.1 g N m^−2^ y^−1^) of labeled N to measure N fixation. The amount added was small enough to have a negligible effect on overall N supply in the ecosystem. The unlabeled N fertilizers in the +10, +15, and +15+P treatments were applied (hand-broadcast) as ammonium nitrate until year 4, when ammonium nitrate was no longer available for purchase in bulk in the New York region, at which point urea was used instead. The ammonium nitrate purchased in Hawaii was coated with dolomite; therefore, we added complementary amounts of dolomite to the control and +10 plots to ensure that all plots received the same amounts of dolomite. The P fertilizer was hand-broadcast as monosodium phosphate. All fertilizers were applied four times per growing season (New York) or year (Hawaii). See [62] for further details.

Due to mortality and morbidity during the experiment, a subset of the trees we originally planted were suitable for informing allometric equations. Additionally, some trees in New York were not harvested to continue another experiment. Overall, we used 12 *Robinia* (2, 3, 4, and 3 in the C, +10, +15, and +15+P treatments, respectively), 16 *Betula* (4, 2, 5, and 5), 31 *Gliricidia* (7, 8, 8, and 8), 29 *Casuarina* (6, 8, 8, and 7), 25 *Psidium* (6, 6, 7, and 6), 19 *Acacia* (7, 4, 4, and 4), 26 *Morella* (7, 7, 5, and 7), and 22 *Dodonaea* (6, 6, 6, and 4) trees to develop all allometric equations for *Robinia* and *Betula* and the allometric equations for aboveground biomass for the species grown in Hawaii. For allometric equations that modeled the biomass components (foliage, twig, secondary stem, and main stem) of the species grown in Hawaii, the goal was to use a sample size of at least 3 trees per treatment for each species of N-fixing trees. However, due to time constraints, fewer than 3 *Psidium* and *Dodonaea* were separated into biomass components. Sample sizes for overall biomass are listed in Table 1 and sample sizes for individuals split into discrete biomass components are listed in Tables 2 and 3.

**Table 1:** Medians, ranges, and sample sizes (n) of size metrics.

	Basal diameter (cm)	Height (m)	Canopy area^†^ (m^2^)	Aboveground Biomass (kg)	Belowground Biomass (kg)	n
*Robinia pseudoacacia* *	9.20 (2.64–15.0)	5.47 (1.38–7.77)	14.4 (0.696–32.9)	14.9 (0.219–43.5)	4.17 (0.133–13.0)	12
*Betula nigra*	5.27 (1.61–14.7)	2.75 (1.08–6.74)	2.33 (0.208–19.3)	1.40 (0.0480–16.1)	0.527 (0.0279–5.71)	16
*Gliricidia sepium* *	3.54 (0.847–11.2)	1.04 (0.240–2.40)	3.65 (0.0204–47.1)	0.549 (0.00340–26.3)	NA	31
*Casuarina equisetifolia* *	8.28 (2.48–15.2)	4.70 (1.42–8.40)	11.0 (0.140–39.3)	5.65 (0.158–23.0)	NA	29
*Psidium cattleianum*	3.31 (1.26–6.22)	0.790 (0.390–1.89)	1.12 (0.160–3.12)	0.413 (0.0273–2.34)	NA	25
*Acacia koa* *	8.57 (1.79–16.4)	3.70 (1.05–5.30)	5.74 (0.260–22.8)	6.45 (0.0874–55.5)	NA	19
*Morella faya* *	5.37 (1.71–10.5)	2.35 (0.510–5.00)	2.39 (0.133–18.6)	3.14 (0.0345–30.3)	NA	26
*Dodonaea viscosa*	6.47 (0.905–13.3)	2.51 (0.910–3.55)	5.94 (0.0992–15.9)	3.58 (0.0187–20.6)	NA	22

*Nitrogen-fixing species

^†^Canopy area defined as maximum canopy width multiplied by maximum canopy length (not multiplied by π)

**Table 2:** Medians, ranges, and sample sizes (n) of biomass components (dry mass).

	Leaf biomass (kg)	Twig Biomass (kg)	Secondary Stem Biomass (kg)	Main Stem Biomass (kg)	n
*Robinia pseudoacacia* *	1.62 (0.0831–5.72)	2.20 (0.0382–6.82)	4.11 (0.0770–13.5)	4.45 (0.104–15.6)	12
*Betula nigra*	0.171 (0.000975–1.53)	0.324 (0.0217–3.43)	0.0983 (0–2.51)^†^	0.53 (0.0254–9.74)	16
*Gliricidia sepium* *	0.115 (0.0003–4.43)	0.0285 (0.0001–1.49)	NA^@^	0.334 (0.0026–20.5)^@^	17
*Casuarina equisetifolia* *	1.04 (0.0365–8.85)	1.01 (0.0518–3.39)	0.549 (0–2.84)^†^	2.57 (0.0644–9.34)	12
*Psidium cattleianum*	0.252 (0.152–0.477)	0.160 (0.117–0.495)	NA^@^	0.113 (0.0553–0.832)^@^	3
*Acacia koa* *	1.54 (0.0452–10.3)	0.436 (0.00640–4.31)	0.754 (0–29.3)^†^	1.54 (0.0358–10.8)	11
*Morella faya* *	0.886 (0.0205–8.64)	0.543 (0.00340–5.19)	NA^@^	1.06 (0.0106–16.5)^@^	13
*Dodonaea viscosa*	0.798 (0.0726–3.27)	0.613 (0.0395–3.66)	NA^@^	2.51 (0.111–7.51)^@^	6

*Nitrogen-fixing species

^†^Given the definition of secondary stems (>1 cm diameter), some trees had no secondary stems, as all stem material was either main stem or twig

^@^Stem material was not separated into secondary and main stem for *Gliricidia*, *Psidium*, *Morella*, or *Dodonaea*

**Table 3:** Means, ranges, and sample sizes (n) of biomass components as proportions of their sum (aboveground biomass, not including fruits).

	Leaf biomass (%)	Twig Biomass (%)	Stem Biomass (%)	n
*Robinia pseudoacacia* *	12.6 (5.4–23.2)	18.4 (10.4–41.4)	69.0 (45.5–82.5)	12
*Betula nigra*	10.7 (0.7–21.4)	34.8 (15.3–64.3)	54.5 (27.9–77.7)	16
*Gliricidia sepium* *	25.9 (6.8–68.4)	6.3 (2.3–12.4)	67.8 (26.5–90.9)	17
*Casuarina equisetifolia* *	27.3 (9.0–49.2)	19.8 (12.6–32.8)	52.9 (24.8–69.1)	12
*Psidium cattleianum*	40.4 (26.4–48.0)	31.3 (27.5–36.1)	28.2 (17.0–46.1)	3
*Acacia koa* *	29.5 (15.9–51.7)	9.0 (4.7–17.5)	61.6 (41.0–78.2)	11
*Morella faya* *	42.8 (28.5–63.1)	20.6 (9.9–27.8)	36.6 (21.5–54.4)	13
*Dodonaea viscosa*	25.8 (11.6–35.5)	19.7 (14.9–25.3)	54.5 (39.7–73.5)	6

*Nitrogen-fixing species

### 2.3 Biomass estimates

We harvested the trees planted at our New York site in October 2019 after a five-year growth period. We harvested the trees planted at the Hawaii sites in July 2019 after a four-year growth period. Immediately prior to harvest, we measured stem basal diameter, maximum tree height, canopy length, and canopy width. Stem basal diameter (taken as close to the soil surface as possible) was measured with calipers and reported in cm. We used basal diameter rather than diameter at breast height (1.3 m; which was also measured on some trees) because some individuals were less than 1.3 m tall. For noticeably non-circular stems, we used the geometric mean of the widest diameter and the orthogonal diameter. Tree height (height above the ground, not length of the stem) was measured with a tape measure taped to an extendable pruning pole and reported in m. Canopy length and width, both reported in m, were measured orthogonal to each other with a tape measure. Instead of using canopy length and width separately, we used their product, to which we refer hereafter as canopy area. We note that the actual canopy area would be the product of length, width, and π/4, rather than the product of length and width; therefore, our metric is more precisely “proportional to canopy area” than canopy area itself.

To harvest, we felled trees at the base with a chainsaw (large trees) or hand saw (small trees). For all trees in our New York site and a subset of trees in our Hawaii sites, we separated aboveground tissues into different tissue types in the field: foliage + twigs (branches < 1 cm) and stems (branches ≥ 1 cm). For *Robinia*, *Betula*, *Casuarina*, and *Acacia*, we further separated stems into main stem (the single thickest part of the stem of each branch point until the stem was < 1 cm) and secondary stems (all other stems > 1 cm in diameter). We did not separate stems into main and secondary for *Gliricidia*, *Psidium*, *Morella*, or *Dodonaea*, which have bifurcating stems. Immediately after felling trees, we recorded the mass of each biomass component (or total aboveground biomass for the subset of trees in Hawaii that were not separated into tissue types) in the field using a hanging balance (for biomass components that did not fit on the top-loading field balance) or a top-loading field balance. Representative subsamples of foliage + twigs were taken back to the lab and separated into foliage and twigs, after which representative subsamples of each tissue type (foliage, twigs, secondary stem, and main stem) were oven-dried at 65°C for at least 48 hours. We measured the masses of these dried samples. These wet:dry mass ratios were used to calculate dry mass for each biomass component. All mass values reported herein are dry masses.

We also harvested belowground biomass in New York. A hydraulic mini excavator along with manual digging with a shovel was used to loosen the rooting system from the soil and unearth relatively intact rooting systems. Given the disruptive nature of unearthing entire rooting systems, some fine roots were lost during the harvest. The vast majority of coarse roots, however, were recovered and massed; root systems were reconstructed in the lab and we measured breakages of diameter ≥ 0.5 cm for which the corresponding root was not recovered: of 72 breakages from 28 trees, a majority of breakages had diameters <1.0 cm and all but one—a 3.0 cm breakage from a large *Robinia*—had diameters <2.0 cm. Rooting systems were taken back to the lab, cleaned, air dried for at least 120 days, then measured for mass. As above, representative subsamples of the air-dried rooting systems were oven-dried at 65°C for at least 48 hours, and the wet:dry ratios were used to calculate dry belowground biomass. Unfortunately, due to logistical infeasibility and site restrictions, we did not harvest belowground biomass in Hawaii.

### 2.4 Statistics

Allometric relationships typically follow power laws [65]. Therefore, we used power laws with one or more driver variable(s) per response variable. As allometric driver variables, we used basal diameter (*D*, in cm), tree height (*H*, in m), and canopy area (width multiplied by length; *A*, in m^2^), in addition to the composite variables *D*^2^*H* and *D*^2^*HA*. As treatment driver variables, we used the fertilization treatment (indexed *t*, to indicate separate parameters for the C, +10, +15, and +15+P treatments).

For each species, we compared candidate models that included all reasonable combinations of the allometric and treatment driver variables. For response variables, we used aboveground biomass (*AGB*), belowground biomass (*BGB*), total biomass (*B*), foliar biomass (*FB*), twig biomass (*TwB*), secondary stem biomass (*SSB*, where applicable), and main stem biomass (*MSB*), all in kg. We constructed fits for each of these independently, rather than summing the components of aboveground biomass or summing aboveground and belowground biomass. For example, the simplest equation we used for total biomass, which models total biomass as a function of diameter alone, was

$$B=exp(c)D^{\alpha }\exp\left(\varepsilon \right)$$ (1)

where the exp(*c*) parameter is the expected biomass of a tree with *D* = 1 cm, *α* is the scaling exponent with diameter, and exp(*ε*) is a lognormally-distributed error term. A more complicated model, which models total biomass as a function of the square of diameter multiplied by height, canopy area, and treatment, was

$$B=exp(c_{t}){\left(D^{2}H\right)}^{\gamma_{t}}A^{\delta_{t}}\exp\left(\varepsilon \right)$$ (2)

where the four exp(*c*_*t*_) parameters are the expected biomasses of a tree with *D*^2^*H* = 1 cm^2^·m and *A* = 1 m^2^ in the four different fertilization treatments. The parameters *γ*_*t*_ and *δ*_*t*_ are the scaling exponents for the square of diameter multiplied by height and for canopy area, respectively, both of which vary across the four fertilization treatments.

All variables used in these equations were lognormally distributed, as is common in allometric studies. Therefore, we used log-transformed data for analysis, though we present data in untransformed values (e.g., kg rather than log(kg)). Because we log-transformed data for analysis, we used the log-transformed versions of Eqs 1 and 2:

ln(B)=c+αln(D)+ε (3)

$$\ln\left(B\right)=c_{t}+\gamma_{t}\;\ln\left(D^{2}H\right)+\delta_{t}\;\ln\left(A\right)+\varepsilon$$ (4)

To find the most parsimonious model, we used Akaike’s Information Criteria (AIC) to compare the candidate models [66]. In some cases, the best fit according to AIC was overfitted to the data. In these cases, we removed the overfitted models from the set of candidate models. We report up to four separate models for each combination of species and response variable: the most parsimonious model overall, the most parsimonious model without a treatment effect, the most parsimonious model without canopy area (because diameter and height are more commonly measured), and the model with diameter as the only driver (because of the wider availability of data on diameter than on height or canopy area).

Although our primary focus was to establish the best allometric relationships for each of these species, we also addressed our secondary questions about the effects of fertilization and functional type (N-fixer vs. non-fixer) on the allometric relationships of these species. To assess the effects of fertilization, we examined whether the best fit for each species included treatment (Question 1a). If there was no observable effect, we concluded that fertilization did not have an effect. Alternately, if treatment did have an effect, we assessed consistency across treatment types. If the fertilization effects were consistent across treatments (e.g., the +15 treatment had a similar or greater effect than the +10 treatment), we concluded that fertilization had an effect. However, if the best fit model included a treatment effect but the effects were inconsistent across treatment (e.g., if the +15 treatment were more similar to the control than to the +10 treatment), we concluded that fertilization did not have an effect. To assess the degree to which N-fixers and non-fixers responded differently to fertilization treatments, we compared the species-level results across functional type (Question 1b).

To assess the effect of functional type on the allometric relationships (Question 2), we compared AIC values of species-level fits to functional type-level fits. For these comparisons we focused on response variables for which we had data across all species: aboveground biomass and the fractions of aboveground biomass comprised of leaves, twigs, and stems (secondary stems and main stems combined). For the species-level vs. functional type (N-fixer vs. non-fixer) comparisons we used basal diameter only as the driver variable.

### 2.5 Comparisons to other data sets

We compared our allometric equations against published equations calculated for some of the same species at other study sites. We found published studies for *Robinia*, *Gliricidia*, and *Casuarina* at similar ages and sizes [25, 67, 68]. We did not find comparable published equations for the rest of the species we studied. Due to differences in the height at which diameter was measured, our allometric equations were not always directly comparable to previously published equations. For example, we measured basal diameter at ground level, whereas some studies measured “basal” diameter at 10 cm [25] or 15 cm [67] above the ground, and others measured diameter at breast height [68] (DBH; diameter at 130 cm above the ground).

To facilitate comparisons to other studies, we assumed that diameter tapers exponentially with height above the base. We measured diameters at multiple heights in our *Robinia* trees to determine the degree of this tapering. Because we only had diameter data at multiple heights for *Robinia*, we used the *Robinia*-derived relationship for *Gliricidia* and *Casuarina* as well as for *Robinia*. We suspect that the degree of tapering might differ across species, but we reasoned that an imperfect correction was better than no correction. For our *Robinia* trees, the ratio of DBH to basal diameter was 50.8%, and thus we derived the exponential parameter *c* from the equation 0.508 = exp (−*c*×130). This gave a value of *c* = 0.0052, so we estimated diameter *D*(*h*) at a given height *h* from diameter at the base *D*(0) as *D*(*h*) = *D*(0)×exp (−0.0052×*h*). For example, diameter at 10 cm height of a tree with a basal diameter of 6 cm would be *D*(10) = 6×exp(−0.0052×10) = 5.70 cm.

Next, we compared our equations to those developed by other investigators. For *Robinia*, Böhm *et al*. (2011) [25] reported a fit of $Woody\;biomass=\exp\left(-3.7933+2.8407\;\ln\left(D_{10}\right)\right)$ (where *D*_10_ is diameter at 10 cm above the ground). The equation from Böhm *et al*. [25] modeled aboveground woody biomass, excluding foliage, so to compare to their equation, we summed the best-fit equations for main stem, secondary stem, and twigs (Table 3). For *Gliricidia*, Harrington & Fownes (1993) [67] reported the fit $Woody\;biomass=\exp\left(-4.1289+3.110\;\ln\left(D_{15}\right)\right)$, where *D*_15_ is diameter at 15 cm above the ground. Similar to the equation from Böhm *et al*. [25], the equation from Harrington & Fownes [67] modeled aboveground woody biomass, excluding foliage. Therefore, to compare to their equation, we used our best-fit equation for *Gliricidia* main stem (which includes secondary stems as well; Table 3). For *Casuarina*, Xue *et al*. (2016) [68] reported multiple separate aboveground tissues in their study. Therefore, we summed their individual fits for Trunkbiomass=exp(−3.413+1.884ln(DBH)+0.941ln(H)),Branchbiomass=exp(−2.388+1.128ln(DBH)+0.094ln(H)+1.109ln(CR)), and Foliarbiomass=exp(−1.272+1.965ln(DBH)−0.644ln(H)), where *DBH* is diameter at 130 cm, *H* is height, and *CR* is crown radius (estimated as the average of half the canopy width and half the canopy length). For each of these tissue types, we compared the fits in Xue *et al*. (2016) [68] to best fit equations for *Casuarina* by using diameter, height, and crown dimension data from our trees in their equations vs. in our equations.

## 3. Results

### 3.1 Summary statistics of biomass components

**Fig 1:**
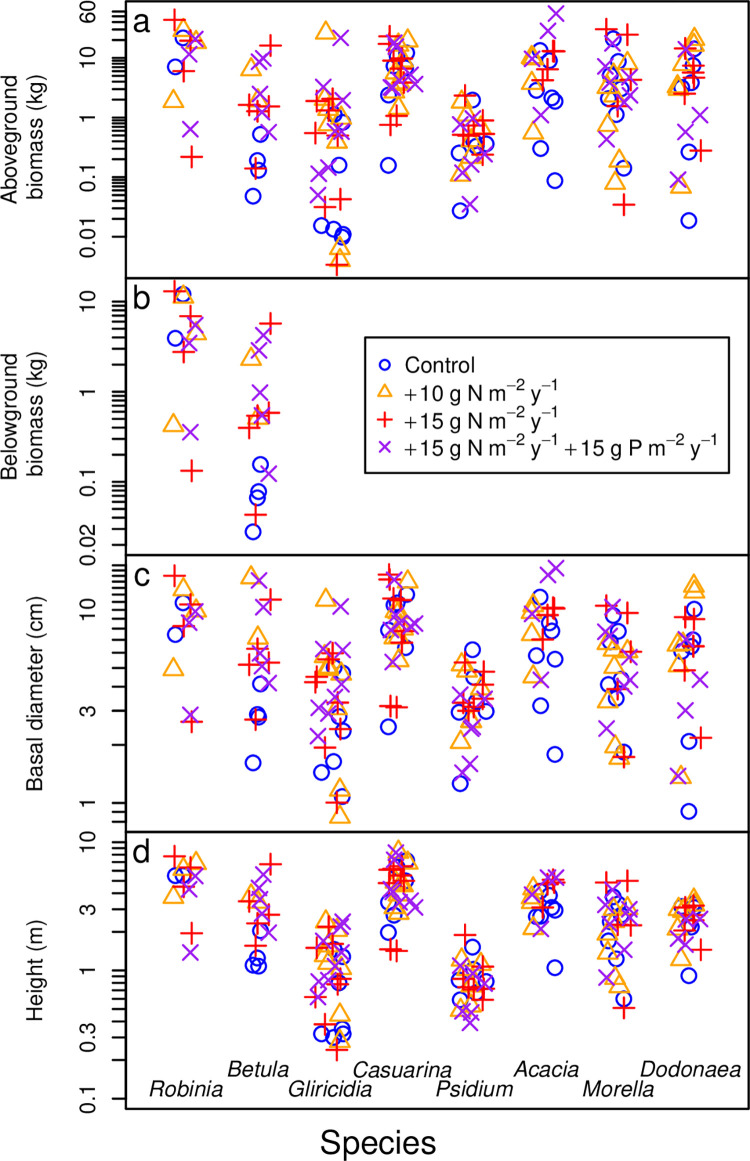
Size distributions of eight tree species. (a) Aboveground biomass, (b) belowground biomass, (c) basal diameter, and (d) height are shown for each species. Each symbol represents one individual tree. Color and symbol indicate the fertilization treatment, as indicated in the legend. Data were jittered horizontally for visual clarity. Vertical dotted lines separate the points to clarify which points correspond to which species.

**Fig 2:**
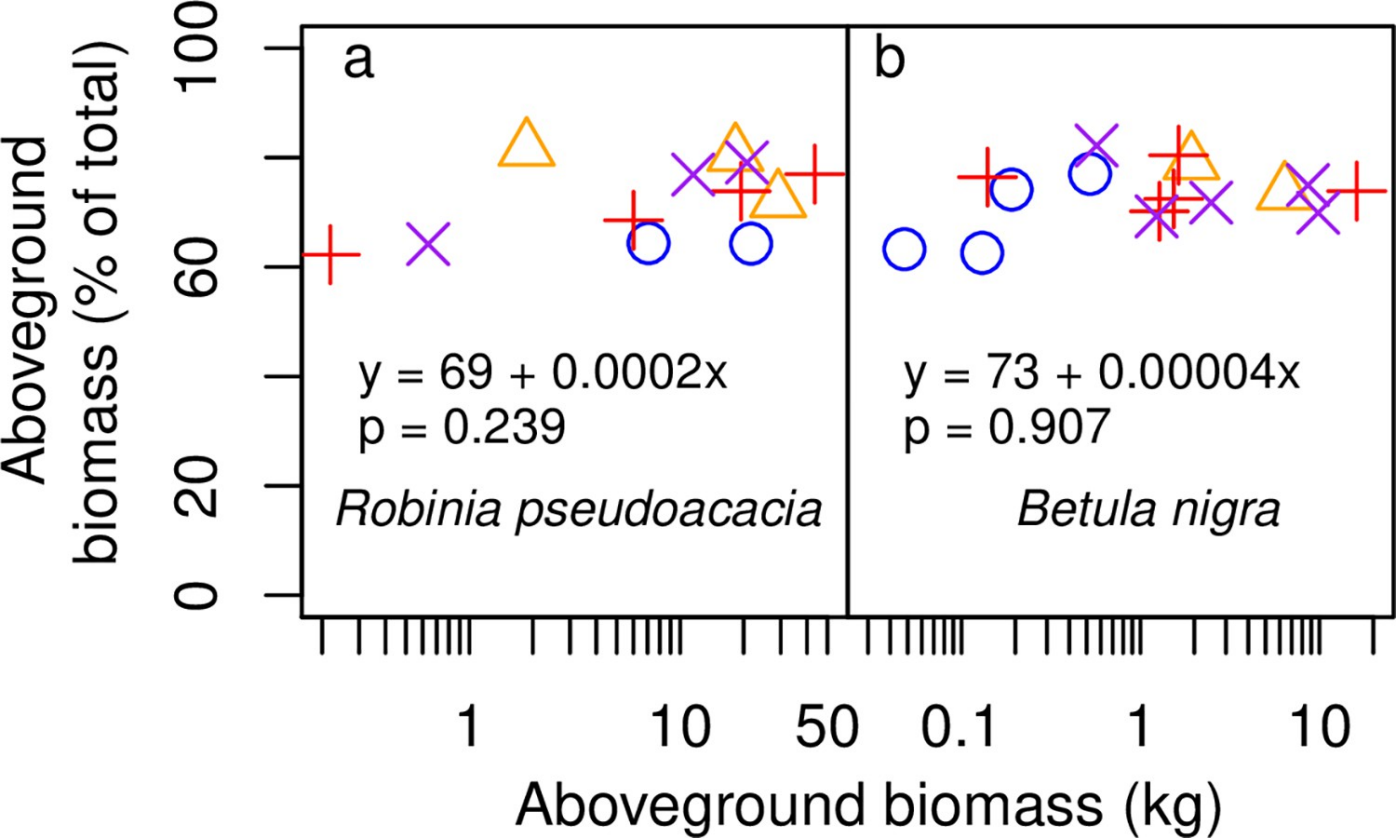
Allocation to aboveground biomass across tree size. (a) *Robinia pseudoacacia* and (b) *Betula nigra*. Each symbol represents an individual tree. Colors and symbols indicate treatments, as in Fig 1. Linear regression equations and p values are shown.

**Fig 3:**
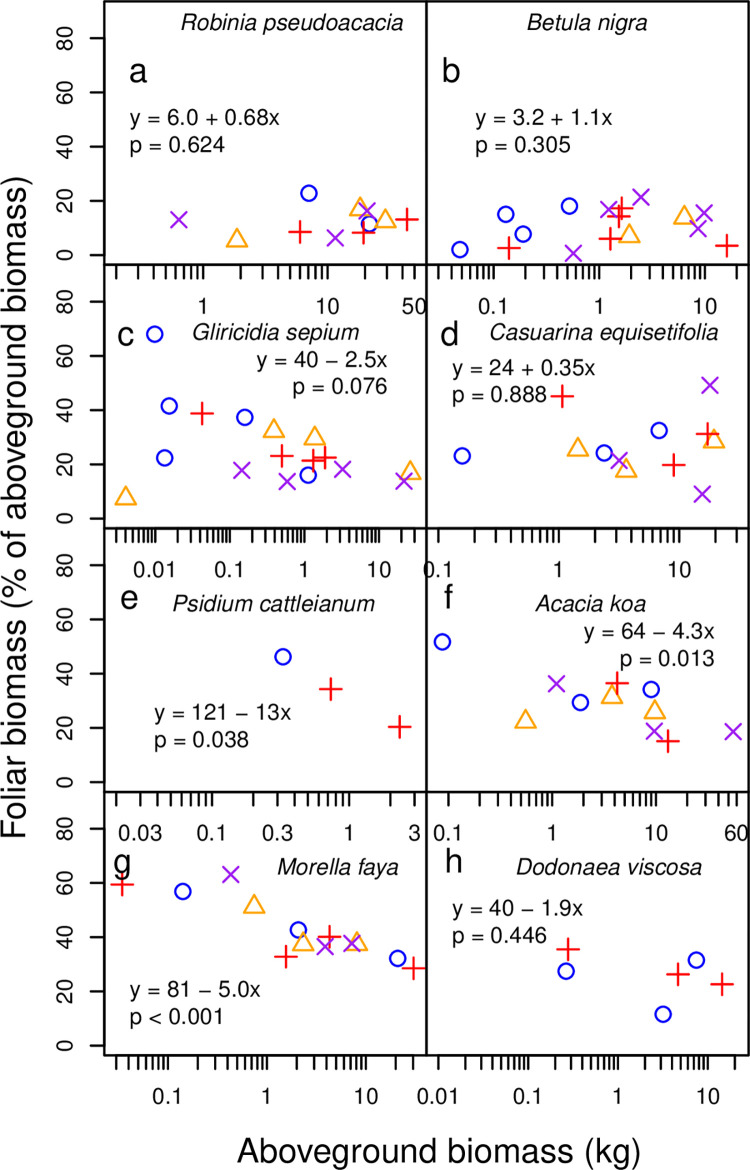
Allocation to foliage across tree size. The fractions of aboveground biomass comprised of foliage are shown for (a) *Robinia pseudoacacia*, (b) *Betula nigra*, (c) *Gliricidia sepium*, (d) *Casuarina equisetifolia*, (e) *Psidium cattleianum*, (f) *Acacia koa*, (g) *Morella faya*, and (h) *Dodonaea viscosa*. Each symbol represents one individual tree. Colors and symbols indicate treatments, as in Fig 1. Linear regression equations and p values are shown.

At harvest time, trees spanned a range of sizes (Fig 1, Table 1). In New York, *Robinia* trees ranged from 0.219–43.5 kg aboveground biomass, 0.133–13.0 kg belowground biomass, 2.64–15.0 cm basal diameter, and 1.38–7.77 m height (Fig 1, Table 1). Aboveground tissues comprised an average (arithmetic mean, which is used hereafter for averages unless noted) and range of 72% (62–82%) of total *Robinia* biomass (Fig 2A). Within aboveground tissues, foliage comprised 12.6% (5.4–23.2%) (Fig 3A), twigs 18.4% (10.4–41.4%), secondary stems 29.2% (13.8–46.7%), and main stem 39.8% (24.4–53.7%) (Tables 2 and 3).

*Betula* trees were smaller than *Robinia* trees on average, ranging from 0.0480–16.1 kg aboveground biomass, 0.0279–5.71 kg belowground biomass, 1.61–14.7 cm basal diameter, and 1.08–6.74 m height (Fig 1, Table 1). The root:shoot ratios of *Betula* were similar to those of *Robinia*: aboveground tissues comprised 73% (63–82%) of total *Betula* biomass (Fig 2B). The breakdown of aboveground tissues was also similar to *Robinia*, except for a higher proportion of twigs and a lower proportion of secondary stem: in *Betula*, foliage comprised 10.7% (0.7–21.4%) (Fig 3B), twigs 34.8% (15.3–64.3%), secondary stems 10.9% (0–29.1%), and main stem 43.7% (18.4–72.6%, Tables 2 and 3).

In Waiakea, *Gliricidia* trees ranged from 0.00340–26.3 kg aboveground biomass, 0.847–11.2 cm basal diameter, and 0.240–2.40 m height (Fig 1, Table 1). Foliage, twigs, and stems (secondary and main combined) comprised 25.9% (6.8–68.4%) (Fig 3C), 6.3% (2.3–12.4%), 67.8% (26.5–90.9%), respectively, of aboveground biomass (Table 2). Individuals of the other N-fixing species at Waiakea, *Casuarina*, ranged from 0.158–23.0 kg aboveground biomass, 2.48–15.2 cm basal diameter, and 1.42–8.40 m height (Fig 1, Table 1). As fractions of aboveground biomass, foliage comprised 27.3% (9.0–49.2%) (Fig 3D), twigs 19.8% (12.6–32.8%), secondary stem 11.4% (0–21.9%), and main stem 41.5% (17.9–51.9%) (Table 2). Individuals of the non-fixing *Psidium* were smaller, ranging from 0.0273–2.34 kg aboveground biomass, 1.26–6.22 cm basal diameter, and 0.390–1.89 m height (Fig 1, Table 1). Foliage, twigs, and stems (secondary and main combined) comprised 40.4% (26.4–48.0%) (Fig 3E), 31.3% (27.5–36.1%), and 28.2% (17.0–46.1%), respectively, of non-fruit aboveground biomass (fruit was more prevalent in *Psidium* than in other species) (Tables 2 and 3).

In Volcano, *Acacia* trees ranged from 0.0874–55.5 kg aboveground biomass, 1.79–16.4 cm basal diameter, and 1.05–5.30 m height (Fig 1, Table 1). Foliage, twigs, secondary stems, and main stems comprised 29.5% (15.9–51.7%) (Fig 3F), 9.0% (4.7–17.5%), 21.6% (0–53.5%), and 39.9% (19.8–71.4%), respectively, of aboveground biomass (Table 2). For the other N-fixing species at Volcano, *Morella*, aboveground biomass ranged from 0.0345–30.3 kg, basal diameter from 1.71–10.5 cm, and height from 0.510–5.00 m (Fig 1, Table 1). As fractions of aboveground biomass, foliage comprised 42.8% (28.5–63.1%) (Fig 3G), twigs 20.6% (9.9–27.8%), and stems (secondary and main combined) 36.6% (21.5–54.4%) (Table 2). The non-fixing *Dodonaea* ranged from 0.0187–20.6 kg aboveground biomass, 0.905–13.3 cm basal diameter, and 0.910–3.55 m height (Fig 1, Table 1). Foliage, twigs, and stems (secondary and main combined) comprised 25.8% (11.6–35.5%) (Fig 3H), 19.7% (14.9–25.3%), and 54.5% (39.7–73.5%), respectively, of aboveground biomass (Table 2).

Aboveground biomass as a fraction of total biomass did not change with tree size (p = 0.239 for *Robinia*, p = 0.907 for *Betula* for aboveground fraction regressed against the logarithm of aboveground biomass) (Fig 2). Foliar biomass as a fraction of aboveground biomass did not change as a function of tree size for most species (p = 0.624 for *Robinia*, p = 0.305 for *Betula*, p = 0.076 for *Gliricidia*, p = 0.888 for *Casuarina*, and p = 0.446 for *Dodonaea*, for foliar biomass fraction regressed against the logarithm of aboveground biomass), but declined in larger trees for *Psidium* (p = 0.038, though note the sample size of 3), *Acacia* (p = 0.013), and *Morella* (p = 0.00009) (Fig 3).

### 3.2 Best fit allometric equations for aboveground biomass

**Fig 4:**
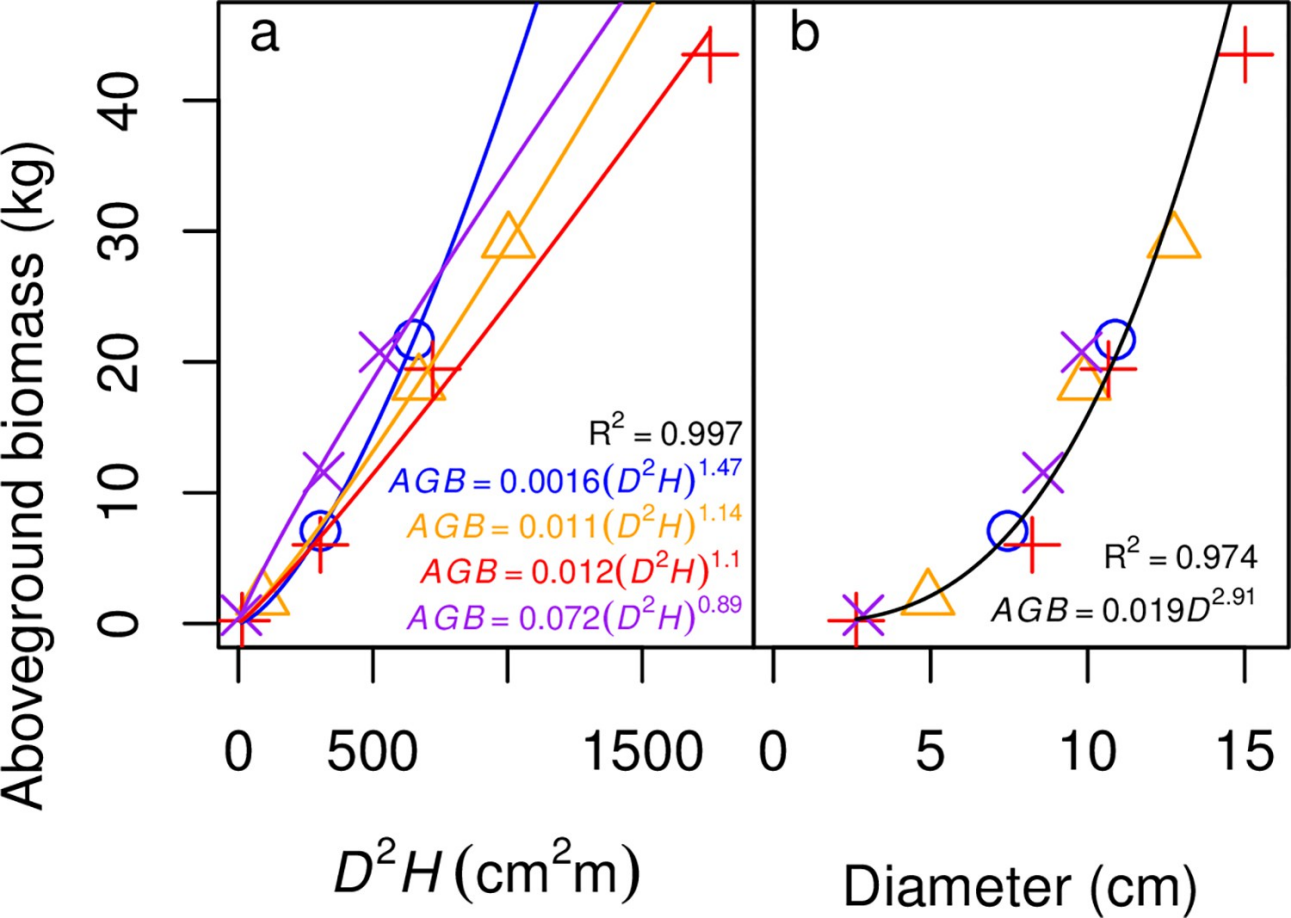
Models for aboveground biomass (AGB) of *Robinia pseudoacacia*. (a) The best fit model according to AIC, which models aboveground biomass as a function of the square of basal diameter (*D*, in cm) multiplied by height (*H*, in m), with different parameters for each treatment. Colors and symbols of the points indicate treatments, as in Fig 1. Colors of curves are analogous: blue is the control; orange is the +10 g N m^−2^ y^−1^ treatment; red is the +15 g N m^−2^ y^−1^ treatment; and purple is the +15 g N m^−2^ y^−1^ +15 g P m^−2^ y^−1^ treatment. (b) Aboveground biomass as a function of basal diameter (*D*) only. Colors and symbols of the points indicate treatments, as in Fig 1. The fit is shown in black because it does not depend on treatment. The fits shown on the panels are the same as in Tables 3 and 6.

The best fit allometric equations were defined as the ones with the lowest AIC score among the candidate models. The best fit allometric equations for aboveground biomass of a number of species, both N-fixers and non-fixers, included treatment effects, with adjusted R^2^ values ranging from 0.92–0.99 (Table 4; hereafter, all R^2^ values reported are adjusted R^2^). Specifically, *Robinia*, *Morella*, and all three non-fixers were best fit by models with treatment effects, whereas *Gliricidia*, *Casuarina*, and *Acacia* were best fit by models without treatment effects (Table 4). The models with treatment effects, however, did not follow our expectations. We would expect the +15 and the +15+P treatments to have a similar or stronger effect on allometric relationships as the +10 treatment. Instead, our results showed that the treatment effects were not consistent. For example, in *Robinia*, the treatment effect for +15+P displayed a higher aboveground biomass relative to diameter^2^ x height, whereas the treatment effects for +15 and +10 displayed lower belowground biomass relative to diameter^2^ x height (Table 4, Fig 4A). In *Morella* and *Betula*, the treatment effect for +15 was more like the control than the treatment effect for +10 (Table 4).

**Table 4:** Best fit models.

Response	Best model	Equation^b^^,^ ^c^	Adj. R^2^	RMSE
Aboveground Biomass, *Robinia pseudoacacia*	*D*^2^*H*×*t*	$AGB=\exp\left(-6.4678\;t_{C}-4.5032\;t_{10}-4.3952\;t_{15}-2.6261\;t_{15+P}+1.4730\;\ln\left(D^{2}H\right)\;t_{C}+1.1393\;\ln\left(D^{2}H\right)\;t_{10}+1.0992\;\ln\left(D^{2}H\right)\;t_{15}+0.8934\;\ln\left(D^{2}H\right)\;t_{15+P}\right)$	0.997	6%
Aboveground Biomass, *Betula nigra*	*D×H+t*	$AGB=\exp\left(-3.9685\;t_{C}-4.5197\;t_{10}-4.1638\;t_{15}-4.1651\;t_{15+P}+1.6614\;\ln\left(D\right)+1.9429\;\ln\left(H\right)-0.1963\;\ln\left(D\right)\;\ln\left(H\right)\right)$	0.989	14%
Aboveground Biomass, *Gliricidia sepium*	*D*^2^*H*×*A*	$AGB=\exp\left(-3.1365+0.4930\;\ln\left(D^{2}H\right)+0.5685\;\ln\left(A\right)+0.0658\;\ln\left(D^{2}H\right)\;\ln\left(A\right)\right)$	0.984	33%
Aboveground Biomass, *Casuarina equisetifolia*	*D*^2^*H*+*A*	$AGB=\exp\left(-2.5574+0.5856\;\ln\left(D^{2}H\right)+0.3754\;\ln\left(A\right)\right)$	0.918	35%
Aboveground Biomass, *Psidium cattleianum*	*D*+*H*+*A×t*	$AGB=\exp\left(-0.5735\;t_{C}-0.6268\;t_{10}-0.2870\;t_{15}-0.6349\;t_{15+P}-0.1764\;\ln\left(D\right)+1.2340\;\ln\left(H\right)+1.2708\;\ln\left(A\right)\;t_{C}+0.9806\;\ln\left(A\right)\;t_{10}+0.5058\;\ln\left(A\right)\;t_{15}+0.6404\;\ln\left(A\right)\;t_{15+P}\right)$	0.928	26%
Aboveground Biomass, *Acacia koa*	*D* ^2^ *HA*	$AGB=\exp\left(-2.7031+0.6224\;\ln\left(D^{2}HA\right)\right)$	0.971	29%
Aboveground Biomass, *Morella faya*	*D*^2^*H*×*A*×*t*	$AGB=\exp(-1.8949\;t_{C}+0.1149\;t_{10}-3.2917\;t_{15}-1.2393\;t_{15+P}+0.6439\;\ln(D_{2}H)\;t_{C}-0.0540\;\ln(D_{2}H)\;t_{10}+1.0310\;\ln(D_{2}H)\;t_{15}+0.4438\;\ln(D_{2}H)\;t_{15+P}+0.6031\;\ln(A)t_{C}+1.3206\;\ln(A)t_{10}+0.2671\;\ln(A)t_{15}+0.6183\;\ln(A)t_{15+P}-0.0140\;\ln(D_{2}H)\ln(A)t_{C}+0.0595\;\ln(D_{2}H)\ln(A)t_{10}-0.0304\;\ln(D_{2}H)\ln(A)t_{15}-0.0054\;\ln(D_{2}H)\ln(A)t_{15+P})$	0.989	12%
Aboveground Biomass, *Dodonaea viscosa*	*D*^2^*HA*×*t*	$AGB=\exp\left(-2.3153\;t_{C}-2.4033\;t_{10}-1.7860\;t_{15}-3.1044\;t_{15+P}+0.6153\;\ln\left(D^{2}HA\right)\;t_{C}+0.5891\;\ln\left(D^{2}HA\right)\;t_{10}+0.5150\;\ln\left(D^{2}HA\right)\;t_{15}+0.7999\;\ln\left(D^{2}HA\right)\;t_{15+P}\right)$	0.987	20%
Belowground Biomass, *Robinia pseudoacacia*	*D*×*t*	$BGB=\exp\left(-4.6027\;t_{C}-6.3219\;t_{10}-4.6041\;t_{15}-3.3184\;t_{15+P}+2.9721\;\ln\left(D\right)\;t_{C}+3.4241\;\ln\left(D\right)\;t_{10}+2.6873\;\ln\left(D\right)\;t_{15}+2.1638\;\ln\left(D\right)\;t_{15+P}\right)$	0.993	7%
Belowground Biomass, *Betula nigra*	*D*+*H*	BGB=exp(−4.5366+1.3177ln(D)+1.4844ln(H))	0.947	41%
Biomass, *Robinia pseudoacacia*	*D*^2^*H*×*t*	$B=\exp\left(-6.0392\;t_{C}-4.4716\;t_{10}-3.8000\;t_{15}-2.0512\;t_{15+P}+1.4751\;\ln\left(D^{2}H\right)\;t_{C}+1.1757\;\ln\left(D^{2}H\right)\;t_{10}+1.0561\;\ln\left(D^{2}H\right)\;t_{15}+0.8392\;\ln\left(D^{2}H\right)\;t_{15+P}\right)$	0.998	6%
Biomass, *Betula nigra*	*D*+*H*+*t*	$B=\exp\left(-3.3913\;t_{C}-3.9491\;t_{10}-3.5645\;t_{15}-3.5502\;t_{15+P}+1.4974\;\ln\left(D\right)+1.5470\;\ln\left(H\right)\right)$	0.986	17%
Foliage, *Robinia pseudoacacia*	*D*	FB=exp(−5.9554+2.8514ln(D))	0.832	70%
Foliage, *Betula nigra*	*DHA*	FB=exp(−9.9883+3.0465ln(D)+3.6711ln(H)−1.3975ln(A))	0.813	130%
Foliage, *Gliricidia sepium*	*D*	FB=exp(−6.5919+3.3388ln(D))	0.935	82%
Foliage, *Casuarina equisetifolia*	*D* ^2^ *HA*	$FB=\exp\left(-3.4694+0.4713\;\ln\left(D^{2}HA\right)\right)$	0.820	79%
Foliage, *Psidium cattleianum*	*D* ^2^ *HA*	$FB=\exp\left(-2.2891+0.3076\;\ln\left(D^{2}HA\right)\right)$	0.802	16%
Foliage, *Acacia koa*	*D* ^2^ *HA*	$FB=\exp\left(-3.3293+0.5255\;\ln\left(D^{2}HA\right)\right)$	0.942	40%
Foliage, *Morella faya*	*D* ^2^ *HA*	$FB=\exp\left(-2.5505+0.5395\;\ln\left(D^{2}HA\right)\right)$	0.964	35%
Foliage, *Dodonaea viscosa*	*D* ^2^ *HA*	$FB=\exp\left(-3.1353+0.4958\;\ln\left(D^{2}HA\right)\right)$	0.870	61%
Twigs, *Robinia pseudoacacia*	*DHA*	TwB=exp(−4.1455+3.4179ln(D)−2.7893ln(H)+0.6997ln(A))	0.951	32%
Twigs, *Betula nigra*	*D* ^2^ *HA*	$TwB=\exp\left(-3.3878+0.4684\;\ln\left(D^{2}HA\right)\right)$	0.942	40%
Twigs, *Gliricidia sepium*	*D*+*H*+*A*	TwB=exp(−7.003+1.9611ln(D)−0.7271ln(H)+0.8040ln(A))	0.950	70%
Twigs, *Casuarina equisetifolia*	*D*^2^*H*×*A*	$TwB=\exp\left(-1.4277-0.2137\;\ln\left(D^{2}H\right)+0.0973\;\ln\left(A\right)+0.1686\;\ln\left(D^{2}H\right)\;\ln\left(A\right)\right)$	0.917	34%
Twigs, *Psidium cattleianum*	*D* ^2^ *HA*	$TwB=\exp\left(-2.8927+0.4290\;\ln\left(D^{2}HA\right)\right)$	0.990	4%
Twigs, *Acacia koa*	*D* ^2^ *HA*	$TwB=\exp\left(-5.0404+0.6076\;\ln\left(D^{2}HA\right)\right)$	0.935	51%
Twigs, *Morella faya*	*D*^2^*H*×*A*	$TwB=\exp\left(-2.9210+0.4098\;\ln\left(D^{2}H\right)+1.4436\;\ln\left(A\right)-0.1092\;\ln\left(D^{2}H\right)\;\ln\left(A\right)\right)$	0.982	27%
Twigs, *Dodonaea viscosa*	*D* ^2^ *HA*	$TwB=\exp\left(-3.6262+0.5445\;\ln\left(D^{2}HA\right)\right)$	0.880	65%
Secondary stem, *Robinia pseudoacacia*	*D*	SSB=exp(−5.7638+3.1684ln(D))	0.947	46%
Secondary stem, *Betula nigra*	*D*	SSB=exp(−6.6658+2.7654ln(D))	0.777	96%
Secondary stem, *Gliricidia sepium*^†^	N/A	N/A	N/A	N/A
Secondary stem, *Casuarina equisetifolia*	*D*+*H*+*A*	SSB=exp(−4.6921+0.2979ln(D)+1.1462ln(H)+0.8321ln(A))	0.980	24%
Secondary stem, *Psidium cattleianum*^†^	N/A	N/A	N/A	N/A
Secondary stem, *Acacia koa*	*D* ^2^ *HA*	$SSB=\exp\left(-7.0019+1.0096\;\ln\left(D^{2}HA\right)\right)$	0.966	34%
Secondary stem, *Morella faya*^†^	N/A	N/A	N/A	N/A
Secondary stem, *Dodonaea viscosa*^†^	N/A	N/A	N/A	N/A
Main stem, *Robinia pseudoacacia*	*D* ^2^ *H*	$MSB=\exp\left(-4.3149+0.9592\;\ln\left(D^{2}H\right)\right)$	0.980	22%
Main stem, *Betula nigra*	*D*+*H*	MSB=exp(−4.0102+0.4005ln(D)+2.6984ln(H))	0.983	23%
Main stem, *Gliricidia sepium*	*D*^2^*H*×*A*	$MSB=\exp\left(-3.5876+0.4966\;\ln\left(D^{2}H\right)+0.6297\;\ln\left(A\right)+0.0607\;\ln\left(D^{2}H\right)\;\ln\left(A\right)\right)$	0.977	44%
Main stem, *Casuarina equisetifolia*	*D* ^2^ *HA*	$MSB=\exp\left(-3.3659+0.5172\;\ln\left(D^{2}HA\right)\right)$	0.938	42%
Main stem, *Psidium cattleianum*	*D* ^2^ *HA*	$MSB=\exp\left(-4.2078+0.7903\;\ln\left(D^{2}HA\right)\right)$	0.970	15%
Main stem, *Acacia koa*	*D*+*H*	MSB=exp(−3.9788+0.8887ln(D)+2.2316ln(H))	0.993	12%
Main stem, *Morella faya*	*D* ^2^ *HA*	$MSB=\exp\left(-3.3604+0.6877\;\ln\left(D^{2}HA\right)\right)$	0.991	20%
Main stem, *Dodonaea viscosa*	*D* ^2^ *H*	$MSB=\exp\left(-4.3164+1.0762\;\ln\left(D^{2}H\right)\right)$	0.971	28%

^a^*D* = Basal diameter in cm; *H* = height in m; *A* = canopy width times canopy length (m^2^); *t* = fertilization treatment, indicating different parameters for control (*t*_C_) vs. +10 g N m^−2^ y^−1^ (*t*_10_) vs. +15 g N m^−2^ y^−1^ (*t*_15_) vs. +15 g N m^−2^ y^−1^ + 15 g P m^−2^ y^−1^ (*t*_15+P_). By definition, the ΔAIC value of each of these models is 0 because each model is the best fit among its candidate set [64].

^b^For models where treatment has an effect, equations are listed with treatments as binary variables. For example, *t*_*c*_ is 1 for the control but 0 for all other treatments.

^c^*AGB* = Aboveground biomass; *BGB* = Belowground biomass; *B* = Biomass; *FB* = Foliar biomass; *TwB* = Twig Biomass; *SSB*: Secondary stem biomass; *MSB* = Main stem biomass.

^†^No secondary stems.

The models for aboveground biomass without a treatment effect had R^2^ values ranging from 0.91–0.99 (Table 5). Restricting the candidate models to only those that use diameter, height, or both (and not canopy area) lowered the goodness of fit for some species, particularly for *Casuarina* (R^2^ = 0.86, down from 0.92) and *Psidium* (R^2^ = 0.87, down from 0.91) (Table 6). Considering diameter as the sole driver lowered the R^2^ of the models with the poorest fits even further, to 0.80 for *Casuarina* and 0.75 for *Psidium*, although the diameter-only model fits for the other species had R^2^ values of at least 0.93 (0.97 for *Robinia* (Fig 4B), 0.93 for *Betula*, 0.95 for *Gliricidia*, 0.96 for *Acacia*, 0.94 for *Morella*, and 0.96 for *Dodonaea*; Table 7).

**Table 5:** Best fit models without a treatment effect.

Response	Best model	ΔAIC*	Equation	Adj. R^2^	RMSE
Aboveground Biomass, *Robinia pseudoacacia*	*D*	23.2	AGB=exp(−3.9401+2.9133ln(D))	0.974	28%
Aboveground Biomass, *Betula nigra*	*D+H*	2.2	$AGB=\exp\left(\begin{array}{c} -3.6218+1.2764\;\ln\left(D\right)\\ +1.6678\;\ln\left(H\right) \end{array}\right)$	0.987	19%
Aboveground Biomass, *Gliricidia sepium*	*D* ^2^ *H×A*	0.0	$AGB=\exp\left(\begin{array}{c} -3.1365+0.4930\;\ln\left(D^{2}H\right)\\ +0.5685\;\ln\left(A\right)\\ +0.0658\;\ln\left(D^{2}H\right)\;\ln\left(A\right) \end{array}\right)$	0.984	33%
Aboveground Biomass, *Casuarina equisetifolia*	*D* ^2^ *H+A*	0.0	$AGB=\exp\left(-2.5574+0.5856\;\ln\left(D^{2}H\right)+0.3754\;\ln\left(A\right)\right)$	0.918	35%
Aboveground Biomass, *Psidium cattleianum*	*D*+*H*+*A*	0.8	$AGB=\exp\left(\begin{array}{c} -1.3543+0.4487\;\ln\left(D\right)\\ +0.8689\;\ln\left(H\right)\\ +0.7488\;\ln\left(A\right) \end{array}\right)$	0.914	35%
Aboveground Biomass, *Acacia koa*	*D* ^2^ *HA*	0.0	$AGB=\exp\left(-2.7031+0.6224\;\ln\left(D^{2}HA\right)\right)$	0.971	29%
Aboveground Biomass, *Morella faya*	*D* ^2^ *H×A*	10.5	$AGB=\exp\left(\begin{array}{c} -1.4218+0.4628\;\ln\left(D^{2}H\right)\\ +0.9086\;\ln\left(A\right)\\ -0.0328\;\ln\left(D^{2}H\right)\;\ln\left(A\right) \end{array}\right)$	0.981	25%
Aboveground Biomass, *Dodonaea viscosa*	*D* ^2^ *HA*	10.1	$AGB=\exp\left(-2.3351+0.6033\;\ln\left(D^{2}HA\right)\right)$	0.975	35%
Belowground Biomass, *Robinia pseudoacacia*	*D*+*H*	24.8	$BGB=\exp\left(\begin{array}{c} -4.6764+3.5761\;\ln\left(D\right)\\ -1.0573\;\ln\left(H\right) \end{array}\right)$	0.945	36%
Belowground Biomass, *Betula nigra*	*D*+*H*	0.0	$BGB=\exp\left(\begin{array}{c} -4.5366+1.3177\;\ln\left(D\right)\\ +1.4844\;\ln\left(H\right) \end{array}\right)$	0.947	41%
Biomass, *Robinia pseudoacacia*	*D*	23.1	B=exp(−3.4358+2.8289ln(D))	0.973	27%
Biomass, *Betula nigra*	*D*+*H*	2.1	$B=\exp\left(\begin{array}{c} -3.2657+1.2818\;\ln\left(D\right)\\ +1.6161\;\ln\left(H\right) \end{array}\right)$	0.982	22%

*Difference in AIC value from the best fit model shown in Table 4. A ΔAIC value greater than 2 is roughly analogous to a significantly worse fit [64].

**Table 6:** Best fit models without canopy area.

Response	Best model	ΔAIC*	Equation	Adj. R^2^	RMSE
Aboveground Biomass, *Robinia pseudoacacia*	*D* ^2^ *H×t*	0.0	$AGB=\exp\left(\begin{array}{c} -6.4678\;t_{C}-4.5032\;t_{10}\\ -4.3952\;t_{15}\\ -2.6261\;t_{15+P}+1.4730\;\ln\left(D^{2}H\right)\;t_{C}\\ +1.1393\;\ln\left(D^{2}H\right)\;t_{10}\\ +1.0992\;\ln\left(D^{2}H\right)\;t_{15}\\ +0.8934\;\ln\left(D^{2}H\right)\;t_{15+P} \end{array}\right)$	0.997	6%
Aboveground Biomass, *Betula nigra*	*D*×*H+t*	0.0	$AGB=\exp\left(\begin{array}{c} -3.9685\;t_{C}-4.5197\;t_{10}\\ -4.1638\;t_{15}-4.1651\;t_{15+P}\\ +1.6614\ln\left(D\right)+1.9429\;\ln\left(H\right)\\ -0.1963\;\ln\left(D\right)\;\ln\left(H\right) \end{array}\right)$	0.989	14%
Aboveground Biomass, *Gliricidia sepium*	*D*+*H*	28.0	$AGB=\exp\left(\begin{array}{c} -4.9621+2.8713\;\ln\left(D\right)\\ +0.7603\;\ln\left(H\right) \end{array}\right)$	0.960	59%
Aboveground Biomass, *Casuarina equisetifolia*	*D* ^2^ *H*	15.0	$AGB=\exp\left(-2.8348+0.7920\;\ln\left(D^{2}H\right)\right)$	0.858	49%
Aboveground Biomass, *Psidium cattleianum*	*D*×*H*×*t*	13.7	$AGB=\exp(\begin{array}{c} -3.6923\;t_{C}-2.4765\;t_{10}-0.0311\;t_{15}\\ +2.2819\;t_{15+P}+2.2127\;\ln(D)t_{C}\\ +1.6752\;\ln(D)t_{10}-0.1103\;\ln(D)t_{15}\\ -2.2968\;\ln(D)t_{15+P}+0.6506\;\ln(H)t_{C}\\ +1.7694\;\ln(H)t_{10}+2.0988\;\ln(H)t_{15}\\ +4.1801\;\ln(H)t_{15+P}\\ -0.1548\;\ln(D)\ln(H)t_{C}\\ -0.7307\;\ln(D)\ln(H)t_{10}\\ -0.2546\;\ln(D)\ln(H)t_{15}\\ -3.5294\;\ln(D)\ln(H)t_{15+P} \end{array})$	0.871	27%
Aboveground Biomass, *Acacia koa*	*D*	6.2	AGB=exp(−4.3772+2.8949ln(D))	0.960	35%
Aboveground Biomass, *Morella faya*	*D* ^2^ *H×t*	22.9	$AGB=\exp\left(\begin{array}{c} -2.5148\;t_{C}-3.0744\;t_{10}-3.7519\;t_{15}\\ -2.1016\;t_{15+P}+0.9225\;\ln\left(D^{2}H\right)\;t_{C}\\ +0.9995\;\ln\left(D^{2}H\right)\;t_{10}+1.1491\;\ln\left(D^{2}H\right)\;t_{15}\\ +0.7833\;\ln\left(D^{2}H\right)\;t_{15+P} \end{array}\right)$	0.972	27%
Aboveground Biomass, *Dodonaea viscosa*	*D* ^2^ *H*	18.8	$AGB=\exp\left(-3.5477+1.0748\;\ln\left(D^{2}H\right)\right)$	0.963	44%
Belowground Biomass, *Robinia pseudoacacia*	*D*×*t*	0.0	$BGB=\exp\left(\begin{array}{c} -4.6027\;t_{C}-6.3219\;t_{10}\\ -4.6041\;t_{15}-3.3184\;t_{15+P}\\ +2.9721\;\ln\left(D\right)\;t_{C}+3.4241\;\ln\left(D\right)\;t_{10}\\ +2.6873\;\ln\left(D\right)\;t_{15}\\ +2.1638\;\ln\left(D\right)\;t_{15+P} \end{array}\right)$	0.993	7%
Belowground Biomass, *Betula nigra*	*D*+*H*	0.0	$BGB=\exp\left(\begin{array}{c} -4.5366+1.3177\;\ln\left(D\right)\\ +1.4844\;\ln\left(H\right) \end{array}\right)$	0.947	41%
Biomass, *Robinia pseudoacacia*	*D*^2^*H*×*t*	0.0	$B=\exp\left(\begin{array}{c} -6.0392\;t_{C}-4.4716\;t_{10}\\ -3.8000\;t_{15}-2.0512\;t_{15+P}\\ +1.4751\;\ln\left(D^{2}H\right)\;t_{C}+1.1757\;\ln\left(D^{2}H\right)\;t_{10}\\ +1.0561\;\ln\left(D^{2}H\right)\;t_{15}\\ +0.8392\;\ln\left(D^{2}H\right)\;t_{15+P} \end{array}\right)$	0.998	6%
Biomass, *Betula nigra*	*D*+*H*+*t*	0.0	$B=\exp\left(\begin{array}{c} -3.3913\;t_{C}-3.9491\;t_{10}\\ -3.5645\;t_{15}-3.5502\;t_{15+P}\\ +1.4974\;\ln\left(D\right)+1.5470\;\ln\left(H\right) \end{array}\right)$	0.986	17%
Foliage, *Robinia pseudoacacia*	*D*	0.0	FB=exp(−5.9554+2.8514ln(D))	0.832	70%
Foliage, *Betula nigra*	*D* ^2^ *H*	0.5	$FB=\exp\left(-7.2723+1.1196\;\ln\left(D^{2}H\right)\right)$	0.787	161%
Foliage, *Gliricidia sepium*	*D*	0.0	FB=exp(−6.5919+3.3388ln(D))	0.935	82%
Foliage, *Casuarina equisetifolia*	*D*	3.4	FB=exp(−4.4812+2.2615ln(D))	0.761	95%
Foliage, *Psidium cattleianum*	*H*	1.4	FB=exp(−1.3017+0.9011ln(H))	0.687	20%
Foliage, *Acacia koa*	*D*	4.5	FB=exp(−4.8276+2.4939ln(D))	0.912	51%
Foliage, *Morella faya*	*D* ^2^ *H*	7.5	$FB=\exp\left(-3.4734+0.8913\;\ln\left(D^{2}H\right)\right)$	0.936	49%
Foliage, *Dodonaea viscosa*	*D*	1.1	FB=exp(−4.2035+2.2262ln(D))	0.843	69%
Twigs, *Robinia pseudoacacia*	*D*+*H*	4.3	TwB=exp(−5.6296+4.3701ln(D)−2.0512ln(H))	0.925	43%
Twigs, *Betula nigra*	*D* ^2^ *H*	0.3	$TwB=\exp\left(-4.5667+0.8243\;\ln\left(D^{2}H\right)\right)$	0.941	41%
Twigs, *Gliricidia sepium*	*D*	2.5	TwB=exp(−8.3394+3.6327ln(D))	0.937	90%
Twigs, *Casuarina equisetifolia*	*D* ^2^ *H*	11.8	$TwB=\exp\left(-3.8142+0.6573\;\ln\left(D^{2}H\right)\right)$	0.752	75%
Twigs, *Psidium cattleianum*	*H*	4.8	TwB=exp(−1.5145+1.2861ln(H))	0.952	10%
Twigs, *Acacia koa*	D	4.1	TwB=exp(−6.7717+2.8829ln(D))	0.905	65%
Twigs, *Morella faya*	*H*	15.1	TwB=exp(−2.7400+2.9164ln(H))	0.934	64%
Twigs, *Dodonaea viscosa*	*D*	0.7	TwB=exp(−4.8233+2.4597ln(D))	0.866	70%
Secondary stem, *Robinia pseudoacacia*	*D*	0.0	SSB=exp(−5.7638+3.1684ln(D))	0.947	46%
Secondary stem, *Betula nigra*	*D*	0.0	SSB=exp(−6.6658+2.7654ln(D))	0.777	96%
Secondary stem, *Gliricidia sepium*^†^	N/A	N/A	N/A	N/A	N/A
Secondary stem, *Casuarina equisetifolia*	*D* ^2^ *H*	21.8	$SSB=\exp\left(-6.3952+1.0232\;\ln\left(D^{2}H\right)\right)$	0.839	101%
Secondary stem, *Psidium cattleianum*^†^	N/A	N/A	N/A	N/A	N/A
Secondary stem, *Acacia koa*	*D*	7.6	SSB=exp(−8.9775+4.4058ln(D))	0.920	56%
Secondary stem, *Morella faya*^†^	N/A	N/A	N/A	N/A	N/A
Secondary stem, *Dodonaea viscosa*^†^	N/A	N/A	N/A	N/A	N/A
Main stem, *Robinia pseudoacacia*	*D* ^2^ *H*	0.0	$MSB=\exp\left(-4.3149+0.9592\;\ln\left(D^{2}H\right)\right)$	0.980	22%
Main stem, *Betula nigra*	*D*+*H*	0.0	MSB=exp(−4.0102+0.4005ln(D)+2.6984ln(H))	0.983	23%
Main stem, *Gliricidia sepium*	*D*+*H*	6.0	MSB=exp(−5.3859+3.2272ln(D)+0.5510ln(H))	0.965	59%
Main stem, *Casuarina equisetifolia*	*D* ^2^ *H*	5.8	$MSB=\exp\left(-4.3510+0.8910\;\ln\left(D^{2}H\right)\right)$	0.900	56%
Main stem, *Psidium cattleianum*	*H*	3.2	MSB=exp(−1.6690+2.3585ln(H))	0.915	27%
Main stem, *Acacia koa*	*D*+*H*	0.0	MSB=exp(−3.9788+0.8887ln(D)+2.2316ln(H))	0.993	12%
Main stem, *Morella faya*	*D* ^2^ *H*	14.6	$MSB=\exp\left(-4.5590+1.1421\;\ln\left(D^{2}H\right)\right)$	0.973	38%
Main stem, *Dodonaea viscosa*	*D* ^2^ *H*	0.0	$MSB=\exp\left(-4.3164+1.0762\;\ln\left(D^{2}H\right)\right)$	0.971	28%

*Difference in AIC value from the best fit model shown in Table 4. A ΔAIC value of greater than 2 is roughly analogous to a significantly worse fit [64].

^†^No secondary stems.

**Table 7:** Model fits with diameter only.

Response	ΔAIC*	Equation	Adj. R^2^	RMSE
Aboveground Biomass, *Robinia pseudoacacia*	23.2	AGB=exp(−3.9401+2.9133ln(D))	0.974	28%
Aboveground Biomass, *Betula nigra*	28.8	AGB=exp(−4.2841+2.6117ln(D))	0.930	52%
Aboveground Biomass, *Gliricidia sepium*	33.5	AGB=exp(−5.6496+3.6019ln(D))	0.950	69%
Aboveground Biomass, *Casuarina equisetifolia*	24.8	AGB=exp(−2.9969+2.2140ln(D))	0.801	61%
Aboveground Biomass, *Psidium cattleianum*	26.1	AGB=exp(−3.6979+2.3974ln(D))	0.745	72%
Aboveground Biomass, *Acacia koa*	6.2	AGB=exp(−4.3772+2.8949ln(D))	0.960	35%
Aboveground Biomass, *Morella faya*	40.5	AGB=exp(−3.8975+3.0206ln(D))	0.935	53%
Aboveground Biomass, *Dodonaea viscosa*	20.3	AGB=exp(−3.3642+2.6119ln(D))	0.960	46%
Aboveground Biomass, N-fixers	98.6^@^	AGB=exp(−4.7104+3.1323ln(D))	0.907^@^	87%
Aboveground Biomass, non-fixers	98.6^@^	AGB=exp(−3.8048+2.5958ln(D))	0.907^@^	87%
Belowground Biomass, *Robinia pseudoacacia*	25.9	BGB=exp(−4.3616+2.6429ln(D))	0.936	42%
Belowground Biomass, *Betula nigra*	9.2	BGB=exp(−5.1261+2.5061ln(D))	0.902	62%
Biomass, *Robinia pseudoacacia*	23.1	B=exp(−3.4358+2.8289ln(D))	0.973	27%
Biomass, *Betula nigra*	23.8	B=exp(−3.9074+2.5752ln(D))	0.927	53%
Foliage, *Robinia pseudoacacia*	0.0	FB=exp(−5.9554+2.8514ln(D))	0.832	70%
Foliage, *Betula nigra*	2.6	FB=exp(−7.6708+3.1084ln(D))	0.757	179%
Foliage, *Gliricidia sepium*	0.0	FB=exp(−6.5919+3.3388ln(D))	0.935	82%
Foliage, *Casuarina equisetifolia*	3.4	FB=exp(−4.4812+2.2615ln(D))	0.761	95%
Foliage, *Psidium cattleianum*	3.2	FB=exp(−3.5760+1.6700ln(D))	0.430	28%
Foliage, *Acacia koa*	4.5	FB=exp(−4.8276+2.4939ln(D))	0.912	51%
Foliage, *Morella faya*	10.4	FB=exp(−4.4538+2.7857ln(D))	0.920	56%
Foliage, *Dodonaea viscosa*	1.1	FB=exp(−4.2035+2.2262ln(D))	0.843	69%
Twigs, *Robinia pseudoacacia*	9.5	TwB=exp(−5.0187+2.5597ln(D))	0.878	62%
Twigs, *Betula nigra*	2.9	TwB=exp(−4.9089+2.3174ln(D))	0.931	45%
Twigs, *Gliricidia sepium*	2.5	TwB=exp(−8.3394+3.6327ln(D))	0.937	90%
Twigs, *Casuarina equisetifolia*	14.2	TwB=exp(−3.8361+1.7959ln(D))	0.696	86%
Twigs, *Psidium cattleianum*	8.9	TwB=exp(−4.9085+2.4936ln(D))	0.811	21%
Twigs, *Acacia koa*	4.1	TwB=exp(−6.7717+2.8829ln(D))	0.905	65%
Twigs, *Morella faya*	19.7	TwB=exp(−6.0928+3.3777ln(D))	0.906	80%
Twigs, *Dodonaea viscosa*	0.7	TwB=exp(−4.8233+2.4597ln(D))	0.866	70%
Secondary stem, *Robinia pseudoacacia*	0.0	SSB=exp(−5.7638+3.1684ln(D))	0.947	46%
Secondary stem, *Betula nigra*	0.0	SSB=exp(−6.6658+2.7654ln(D))	0.777	96%
Secondary stem, *Gliricidia sepium*^†^	N/A	N/A	N/A	N/A
Secondary stem, *Casuarina equisetifolia*	24.1	SSB=exp(−6.5568+2.8451ln(D))	0.802	116%
Secondary stem, *Psidium cattleianum*^†^	N/A	N/A	N/A	N/A
Secondary stem, *Acacia koa*	7.6	SSB=exp(−8.9775+4.4058ln(D))	0.920	56%
Secondary stem, *Morella faya*^†^	N/A	N/A	N/A	N/A
Secondary stem, *Dodonaea viscosa*^†^	N/A	N/A	N/A	N/A
Main stem, *Robinia pseudoacacia*	0.6	MSB=exp(−4.6278+2.7784ln(D))	0.979	23%
Main stem, *Betula nigra*	35.1	MSB=exp(−5.0817+2.5608ln(D))	0.840	92%
Main stem, *Gliricidia sepium*	6.2	MSB=exp(−5.9610+3.6987ln(D))	0.963	64%
Main stem, *Casuarina equisetifolia*	10.5	MSB=exp(−4.4247+2.4560ln(D))	0.851	72%
Main stem, *Psidium cattleianum*	6.5	MSB=exp(−7.8400+4.5330ln(D))	0.746	51%
Main stem, *Acacia koa*	12.7	MSB=exp(−4.7964+2.6322ln(D))	0.977	24%
Main stem, *Morella faya*	22.3	MSB=exp(−5.8015+3.5608ln(D))	0.952	54%
Main stem, *Dodonaea viscosa*	4.0	MSB=exp(−3.9096+2.5310ln(D))	0.943	41%

*Difference in AIC value from the best fit model shown in Table 4. A ΔAIC value of greater than 2 is roughly analogous to a significantly worse fit [64].

^†^No secondary stems.

^@^ΔAIC values for the N-fixer and non-fixer fits are for the comparison between the functional type model and the species-level model for all trees rather than the fits with other drivers, as is the case for the fits for each species. Similarly, the adjusted R^2^ values for the N-fixer and non-fixer fits are for the model with a functional type effect for all trees.

**Fig 5:**
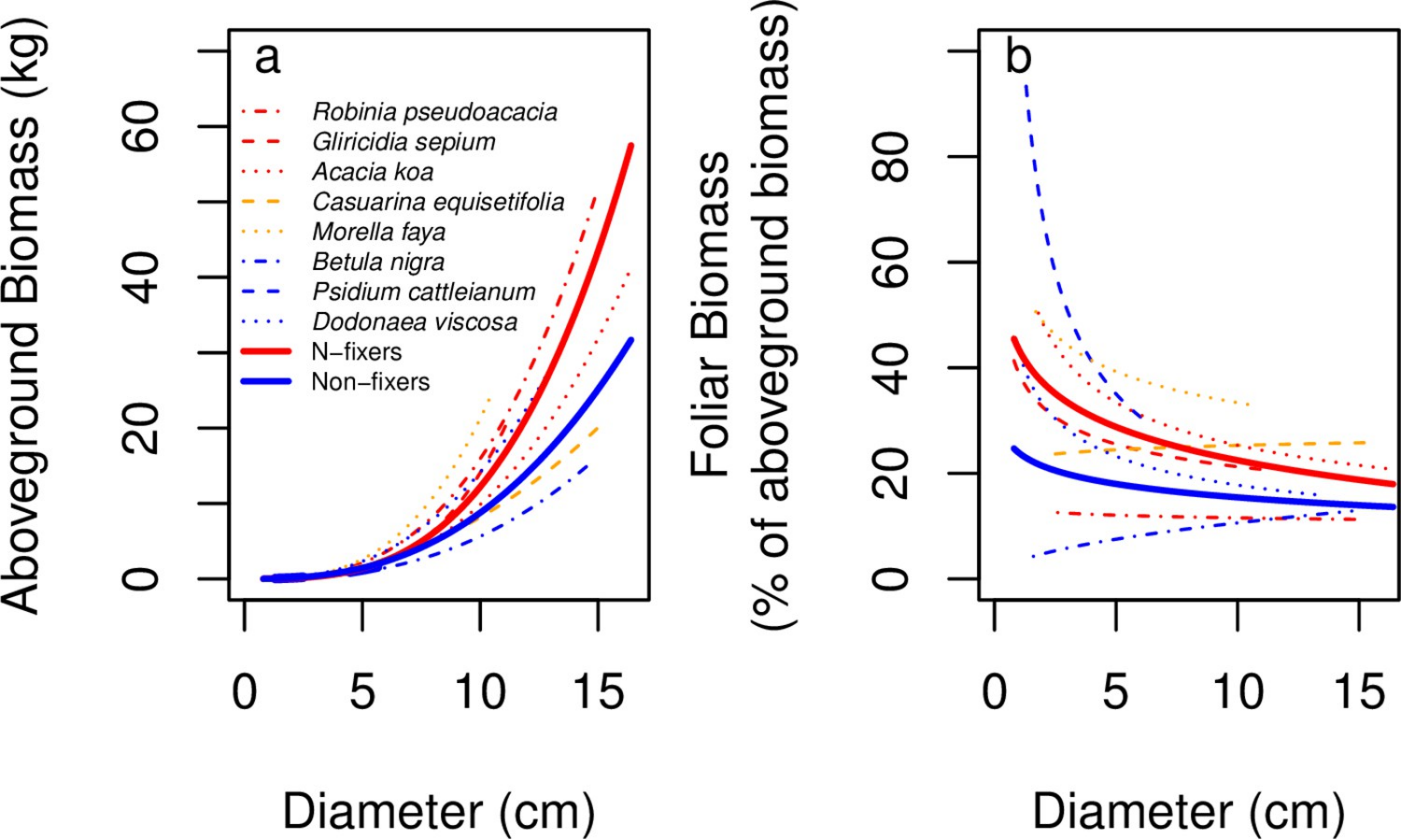
Diameter-driven allometric relationships of species and functional types (Nitrogen-fixing vs. non-fixing tree species). (a) Aboveground biomass is plotted as a function of diameter (*D*, in cm) (b) Foliar biomass is plotted as a function of diameter.

Ultimately, species was a stronger predictor than functional group of the relationship between diameter and aboveground biomass. The fit with species as a driver was stronger, with an overall adjusted R^2^ of 0.951. The fit with functional groups of N-fixers and non-fixers had an adjusted R^2^ of 0.907 and was 98.6 AIC units weaker (Table 7). The functional group model showed that non-fixers accrued less biomass than N-fixing trees for a given basal diameter, but the spread across species within each functional type was large enough that species was a stronger predictor than functional group (Fig 5A).

### 3.3 Best fit allometric equations for belowground and total biomass

The best fit allometric equations for belowground biomass included diameter and treatment for *Robinia* and diameter and height for *Betula*. However, as was the case for aboveground biomass, the treatment effects were not what we would expect from fertilization: the +15 treatment effect was more similar to the control treatment effect than to the +10 treatment effect (Table 4). The fits for total biomass for *Robinia* and *Betula* were similar to those for aboveground biomass (Table 4). The fits without treatment as a possible driver had R^2^ values of 0.95 for belowground biomass for both species, 0.97 for total *Robinia* biomass, and 0.98 for total *Betula* biomass (Table 5). The fits with diameter alone had R^2^ values of 0.94 and 0.90 for belowground biomass of *Robinia* and *Betula*, respectively, and 0.97 and 0.93 for total *Robinia* and *Betula* biomass, respectively (Table 7).

### 3.4 Best fit allometric equations for components of aboveground biomass

Because of the lower sample sizes for components of aboveground biomass (foliage, twigs, secondary stems, and main stems), and because the models with treatment effects gave results that were inconsistent with our expectations for treatment effects (as explained above), we only considered models without treatment effects for the components of aboveground biomass for all species. Foliar biomass was best predicted by a combination of diameter, height, and canopy area for six of the eight species, and by diameter alone in the other two species, with R^2^ values ranging from 0.802 (*Casuarina*) to 0.964 (*Morella*) (Table 4). Removing canopy area as a predictor lowered the R^2^ values (for example, from 0.964 to 0.936 for *Morella* but from 0.802 to 0.687 for *Psidium*) (Table 6). Similarly, using diameter as the only predictor further lowered the R^2^ values (e.g., to 0.920 for *Morella* and to 0.430 for *Psidium*) (Table 7).

Twig biomass was best predicted by a combination of diameter, height, and canopy area for all eight species, with R^2^ values ranging from 0.880 (*Dodonaea*) to 0.990 (*Psidium*) (Table 4). Removing canopy area as a predictor of twig biomass (Table 6) or using diameter as the only predictor of twig biomass (Table 7) typically did not lower the R^2^ as much as for foliar biomass. Similarly, removing canopy area as a predictor of main stem and secondary stem biomass (Table 6) or using diameter as the only predictor for main stem and secondary stem biomass (Table 7) did not lower the R^2^ as much as it did for foliar biomass.

For each of the biomass components for which we could examine the effect of functional type, functional type was not as parsimonious a predictor as species. Leaf biomass as a fraction of aboveground biomass was explained by species, diameter, and the species*diameter interaction (Adj. R^2^ = 0.617) significantly better than by functional type, diameter, and functional type*diameter (Adj. R^2^ = 0.207, ΔAIC = 54.3). Leaf fraction as a function of diameter varied across species (Fig 5B). Similar to our results for foliar biomass, we found that species-level fits were better than functional-group level fits (both crossed with basal diameter) for twig biomass as a fraction of aboveground biomass (Adj. R^2^ = 0.920 compared to 0.394, ΔAIC = 59.4) and stem biomass as a fraction of aboveground biomass (Adj. R^2^ = 0.963 compared to 0.120, ΔAIC = 49.9).

## 4. Discussion

Overall, our results show that the best fit allometric equations predicted aboveground biomass and the components of aboveground biomass well for trees ranging in size from seedlings to small adults (1–16 cm basal diameter) in eight species of N-fixing and non-fixing trees (including two non-fixer species that can be shrubs as well as trees). Our allometric equations also predicted belowground and total biomass well in the two species for which we had belowground data, *Robinia* and *Betula*. Basal diameter as a sole driver typically fit the data well (R^2^ above 0.9 for many variables), though in most cases, including height and canopy area as additional drivers improved the fit. In some cases, including fertilization treatment improved the model fit for aboveground, belowground, or total biomass, but these fertilization treatment effects were inconsistent. Ultimately, we concluded that fertilization with N and P did not have consistent effects on allometric relationships for any of these species, regardless of whether they were N-fixers or non-fixers. Furthermore, although allometric relationships varied widely across species, they did not consistently differ between N-fixing and non-fixing tree species.

The lack of consistent nutrient effects on allometric relationships in our study adds to a list of studies with similar findings [18], although there are also studies showing that nutrients do affect allometry [8, 16, 17]. There are many possible explanations for the lack of nutrient effects in our trees, from effects of ontogeny to small sample size, but we speculate that nutrient limitation, or more specifically a lack of nutrient limitation, plays a major role. The theory that predicts shifts in allometric relationships assumes that nutrients are a limiting resource [17], whereas most of our species were not limited by N or P [69]. We had expected N limitation in the non-fixers given the low extractable N levels in the control soils (means of 0.13–2.0 μg NO_3_-N g soil^−1^ and 2.3–23.3 μg NH_4_-N g soil^−1^ across the species’ plots [62]), but with the exception of *Betula*, which was N limited, none of our species grew faster with N or N+P fertilization [62, 69]. With no limitation by N or P, the mechanistic argument for allometric shifts is missing, consistent with a lack of a fertilization effect on allometric relationships.

The lack of a consistent difference in the allometric relationships between N-fixers and non-fixers is likely due to two factors. First, similar to fertilization effects, the hypothesized mechanism for a consistent N-fixer vs. non-fixer difference in allometry is differential nutrient limitation, based on differential access to nutrients. Given the lack of N limitation to most species, however, the lack of differences between N-fixers and non-fixers makes sense. The second factor concerns variation across species. As can be seen in our data, individual species vary in their allometric relationships, and even if there were strong nutrient limitation, species-level differences may obscure an effect of functional type.

**Fig 6:**
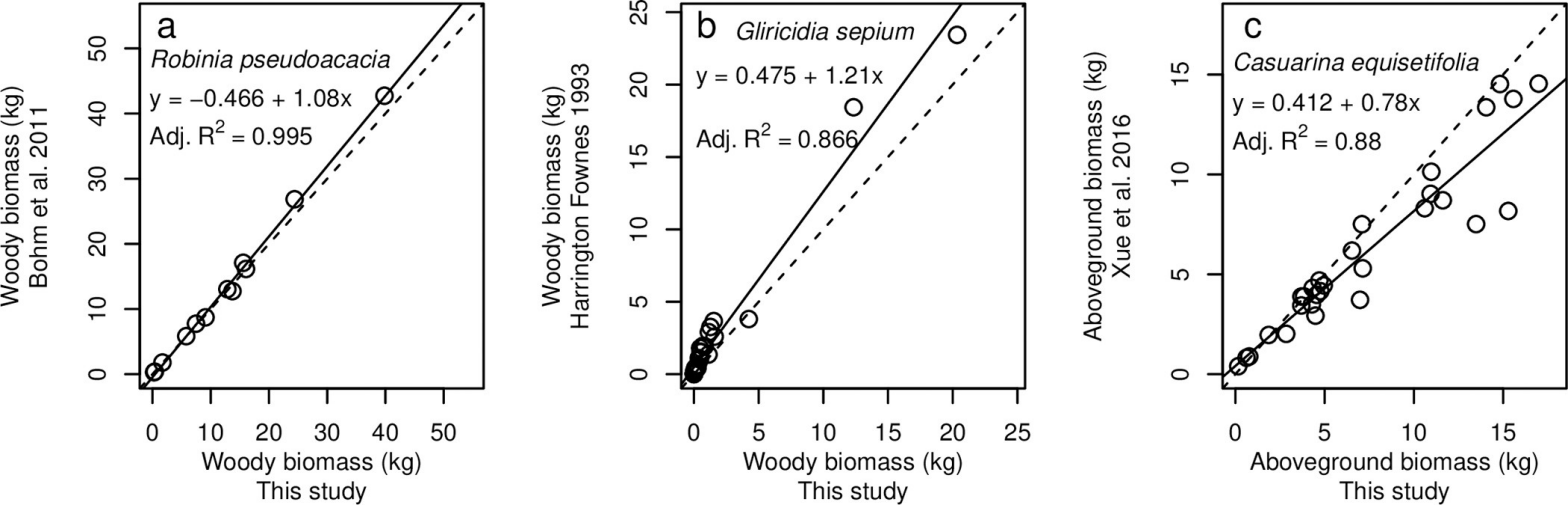
Comparison of our allometric equations to other published equations. We used the input variables (basal diameter, tree height, canopy dimensions) from our trees to estimate biomass components from our equations and from equations from (a) Böhm *et al*. (2011) for *Robinia pseudoacacia*, (b) Harrington & Fownes (1993) for *Gliricidia sepium*, and (c) Xue *et al*. (2016) for *Casuarina equisetifolia*. Each symbol represents one (a) *Robinia pseudoacacia*, (b) *Gliricidia sepium*, or (c) *Casuarina equisetifolia* tree from our dataset. The 1:1 line is plotted in each panel (dotted) along with a linear regression (solid; equations and adjusted R^2^ listed on the figure). See methods for the details of these comparisons.

Our results are comparable to other studies on *Robinia* [25, 70], *Gliricidia* [67], and *Casuarina* [68]; therefore, we sought to compare our allometric equations to published equations. Böhm *et al*. [25] developed allometric equations for aboveground woody biomass (not including foliage) of *Robinia* trees in a similar size range: 0.5–34 kg (compared to 0.22–43.5 kg for our trees). Despite markedly different environmental conditions—their study [25] was in a plantation on a mining reclamation area in Germany, in a drier (560 mm MAP) though similarly cold (9.3°C MAT) climate—the equations from the two studies yielded similar results (Fig 6A). Their allometric equation using diameter to predict aboveground woody biomass fit their data with an R^2^ of 0.91 [25]; whereas our best models for *Robinia* fit our data with R^2^ values of 0.98 for main stem and 0.95 for secondary stem and twigs. After correcting for the different heights of measuring diameter (see methods), our functions (summing main stem, secondary stem, and twigs, but excluding foliage) and their function (of total aboveground woody biomass directly) yielded similar estimates of total aboveground woody biomass for the 12 *Robinia* trees in our study (Fig 6A).

Harrington and Fownes [67] developed allometric equations for aboveground woody biomass (excluding foliage) of *Gliricidia* at four age groups (6, 12, 18, and 24 months after planting) in Maui, Hawaii. The trees used in the Harrington and Fownes study [67] were comparable in size range to the trees used in our study: their diameters ranged between 2.0–8.5 cm after 2 years growth, which falls within the diameter range in our study (0.8–11.2 cm). The allometric equation from Harrington and Fownes [67] that used basal diameter as the only input fit their data with an R^2^ of 0.908, whereas the best fit models of the components of woody biomass from our study had R^2^ values of 0.950 or higher. Using the basal diameters, heights, and canopy dimensions from our *Gliricidia* trees as inputs, the estimates of aboveground biomass from our allometric equation were somewhat lower than estimates from the equation from Harrington and Fownes [67] (i.e., points were above the 1:1 line in Fig 6B).

Xue *et al*. [68] developed allometric equations for biomass components of *Casuarina* for three age ranges, the youngest of which (≤5 years old) was comparable to the *Casuarina* trees in our study (4 years old). Their trees were somewhat larger: 2.5–13.1 cm diameter at breast height (1.3 m above the ground) and 4.1–15.4 m tall compared to 2.5–15.2 cm basal diameter (at ground level) and 1.4–8.4 m tall for our trees. Their study site, on Hainan Island, was at a similar latitude (19.7–20.1°N) to ours (19.6°N). Their R^2^ values for trunk (equivalent to our main stem classification), branch (equivalent to our twig classification), and foliar biomass were 0.994, 0.858, and 0.829 [68], whereas our R^2^ values for main stem, secondary stem, twigs, and foliar biomass were 0.938, 0.980, 0.917, and 0.820 (Table 4). Using the basal diameters, heights, and canopy dimensions from our trees as inputs, the estimates of aboveground biomass from our equation were somewhat higher than estimates from the equation from Xue *et al*. [68] (Fig 6C).

Our equations for *Robinia*, *Gliricidia*, and *Casuarina* gave similar estimates of aboveground biomass as the equations developed by Böhm *et al*. [25], Harrington & Fownes [67], and Xue *et al*. [68]. The small discrepancies we observed could have arisen from a number of possible causes. One possibility is our correction for the different heights at which diameters were measured (see methods). Another possibility is the use of different inputs. For example, our best fits without treatment effects (Tables 3 and 4) often used height and canopy dimensions in addition to diameter, whereas those of Böhm *et al*. [25] and Harrington & Fownes [67] used diameter alone. A third possibility is that the discrepancies arose from real differences in the allometric equations for these species grown in different environmental conditions (i.e., open, high-light versus crowded, shaded forest conditions). Certain species in our experiment (particularly *Gliricidia* and *Psidium*) displayed unexpected growth differences due to the open-light field conditions of our experiment. Normally, these species appear as thin, tall trees crowding together near the forests in Waiakea; however, our trees grew in a short and stocky fashion. We would expect this growth variation to produce differing allometric relationships for aboveground biomass.

Our study is novel in providing multiple allometric equations for each species and each biomass component, each of which might be useful for future studies of these species in the age and size ranges (Table 1) we studied here. For studies of these exact trees in these exact sites, we recommend using the best fits (Tables 4 and 6). Given that the treatment effects were inconsistent with true fertilization effects, though, we recommend that the equations without treatment effects (Tables 5 and 7) be used for these species at other sites. If data on basal diameter, height and canopy area are available, we recommend using the equations in Tables 4 and 5, but if only basal diameter and height or just basal diameter are available, we recommend using the equations in Tables 6 and 7, respectively. Furthermore, our study includes allometric equations for belowground biomass for two species (*Robinia* and *Betula)*, as well as allometric equations for individual tissue components of the eight species we studied, which will help with estimates of total carbon and nutrients for these species.

Species-specific allometric equations can improve estimates of forest carbon stocks and net primary productivity [3, 71]. In selecting N-fixing tree species that are invasive, common, or commonly found in plantations, we aim to improve our ability to estimate biomass and carbon storage. N-fixing trees have often been touted as beneficial for carbon storage [72, 73], though recent work has shown that their carbon benefits can be offset by their stimulation of nitrous oxide emissions [74–76], making accurate estimates of their biomass all the more critical.
